# Case series and finite element analysis of PFNA combined with cerclage wire for treatment of subtrochanteric fracture of femur

**DOI:** 10.1186/s13018-020-02187-3

**Published:** 2021-01-20

**Authors:** Xiaowei Huang, Fangxue Zhang, Yong Zhang

**Affiliations:** 1 Department of Orthopedic Surgery, the First affiliated hospital of Soochow University, No.899, Pinghai Road, Suzhou City, 215000 China; 2 Shanghai Medical College, Fudan University, Shanghai, China

**Keywords:** PFNA, Cerclage wire, Unstable, Subtrochanteric fracture, Clinical efficacy, Finite element analysis

## Abstract

**Objective:**

To retrospectively analyze the clinical efficacy of PFNA combined with a cerclage wire in the treatment of 52 patients with unstable subtrochanteric fracture of the femur and to analyze the biomechanical effect of ligature on a fracture model.

**Methods:**

In this study, 52 patients with unstable subtrochanteric fractures were treated in our orthopedic trauma center from June 2013 to July 2018. The Seinsheimer type IV fracture model was established using the patient’s CT data, and the joint surface of the distal femoral condyle and the external condyle were restrained. The femoral head was used as the loading point, and a force of 500 N was applied vertically along the long axis of the femoral shaft.

**Results:**

All 52 patients were followed up for 12 to 37 months, with an average of 18.07 ± 4.38 months. According to the Sanders hip function score, 28 cases were excellent (55–60 points), 22 cases were good (45–54 points), and 2 cases were poor (35–44 points), with an excellent and good rate of 96.15%. Postoperative deep vein thrombosis occurred in 3 cases, and fracture nonunion occurred in 1 case. No infection, loose fracture of internal fixation or hip varus deformity occurred. The finite element analysis indicated that the displacement of the whole model decreased slightly and the relative sliding of the fracture block decreased, but the maximum stress of the femur increased after the addition of the cerclage wire.

**Conclusion:**

The treatment of unstable subtrochanteric fracture of the femur with PFNA combined with cerclage wire has the advantages of simple operation, satisfactory reduction of fracture, stable fixation, and good recovery of limb function. The finite element analysis suggested that the biomechanical strength fixation was enhanced after the addition of cerclage wire. However, the local stress concentration of the tie may increase the risk of failure.

## Introduction

Intertrochanteric fracture is a common injury in the elderly population. As intertrochanteric bone is mostly cancellous bone, elderly patients are prone to fracture due to brittle bone tissue and weakened tensile strength. However, subtrochanteric fracture of the femur is a special type of peritrochanteric fracture, accounting for 10–30% of all hip fractures [1–3]. They occur not only in elderly osteoporosis patients but also in young and middle-aged patients. Because the inferior trochanteric region of the femur is mainly composed of cortical bones, the blood supply is relatively insufficient compared to the intertrochanteric region [4–6]. Most cases are caused by severe injury violence and often require surgical reduction and internal fixation to minimize the incidence of fracture malunion, nonunion, and coxa varus deformity [7, 8]. The anatomical structure of the subtrochanteric region of the femur is in the region with the most concentrated stress conduction, in which the medial and posterior medial cortical bones bear high compressive stress, while the lateral cortex bears high tension stress [9, 10].

For subtrochanteric fractures of the femur, the medial cortical bone is mostly comminuted, resulting in bilateral mechanical asymmetry. In the case of extramedullary plate and screw system fixation, the moment arm is longer than that in intramedullary fixation [11], with weaker anti-rotation ability and more time for postoperative mobilization. Compared with external medullary fixation, intramedullary fixation can conduct stress more uniformly, which greatly reduces the stress on the internal implant compared with the screw-plate system, thus greatly improving the postoperative stability of fracture and reducing the incidence of complications such as postoperative fracture nonunion and hip varus [12–16].

This study retrospectively analyzed 52 cases of unstable subtrochanteric femoral fractures treated with proximal femur nail anti-rotation combined with cerclage wire and limited open reduction and fixation of fracture ends in the orthopedic trauma center of our hospital from June 2013 to July 2018. In addition, a finite element analysis was utilized to analyze the underlying mechanical mechanism.

## Patients and methods

### Demographic statistics

**Fig. 1:**
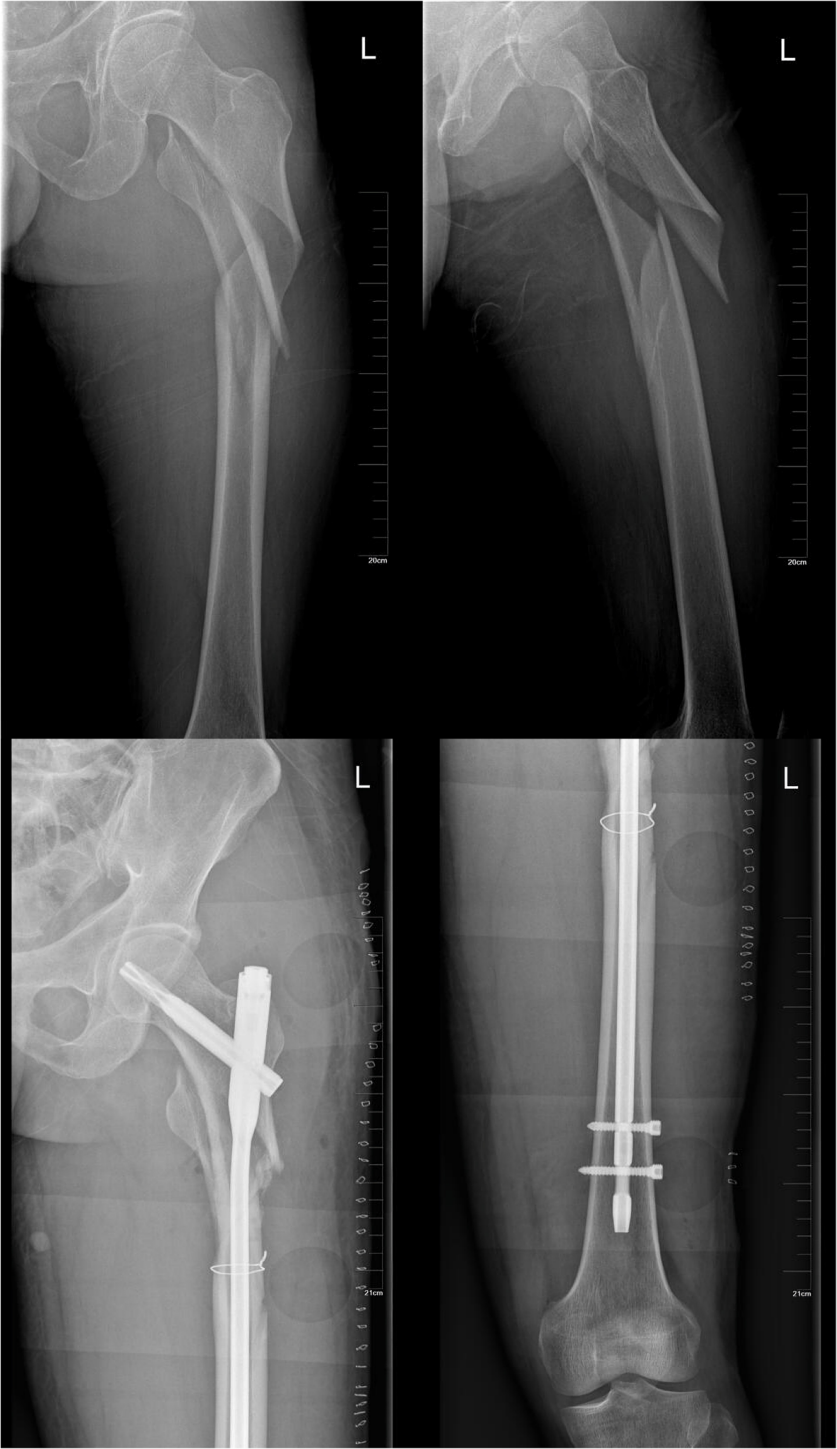
A typical case of a 43-year-old man with left subtrochanteric fracture caused by a car accident was treated with limited open reduction and PFNA combined with cerclage wire

**Fig. 2:**
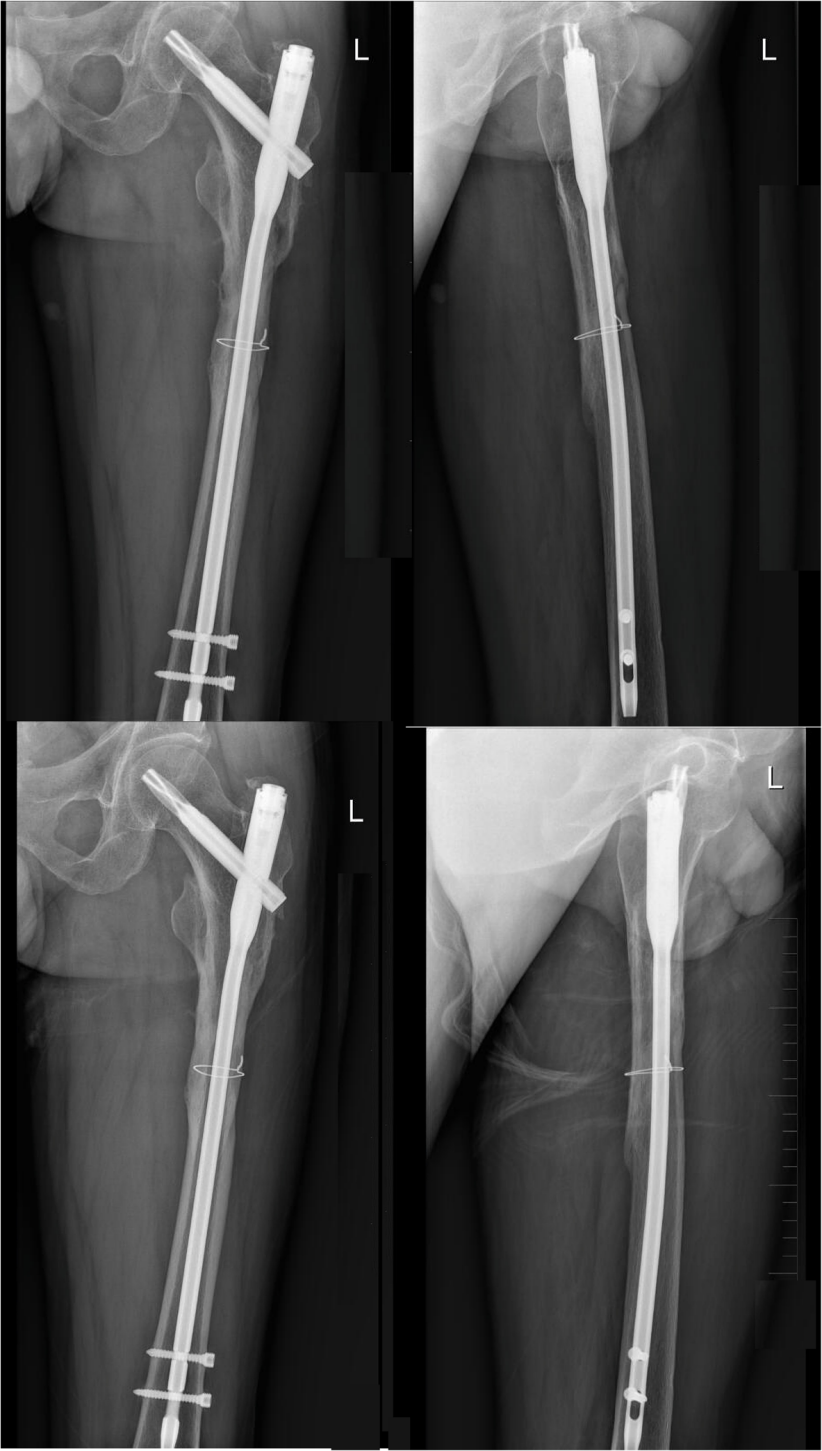
Re-examination 6 months after the operation showed obvious callus formation and fuzzy fracture line (upper). The re-examination 12 months after the operation indicated that the fracture end alignment was good and the fracture had been completely healed. **a**, **b** Preoperative X-ray of the affected hip joint (lower)

Fifty-two patients with subtrochanteric fractures who were admitted to the orthopedic trauma center of our hospital from June 2013 to July 2018 were selected for this study. Patients with acute closed subtrochanteric fracture of the femur, patients without other fractures in the ipsilateral limb, patients with no cerebral infarction or abnormal muscle strength, and patients who tolerated rehabilitation training as well as surgery were included in the study. Patients with pathological fractures, malignant tumors, and patients who were lost to follow-up were excluded. Typical patients are shown in Figs. 1 and 2.

The mean age of the patients was 76.53 ± 6.38 years. There were 35 male cases and 17 female cases included. In 33 cases, the fracture was in the left limb, and in 19 cases the fracture was in the right limb. In 16 cases, patients were injured by traffic accidents. In 36 cases, patients fell from height, resulting in fracture. Regarding complications, there were 2 cases of coronary heart disease, 1 case of gout, 5 cases of hypertension, 2 cases of diabetes, and 3 cases of hypertension with diabetes.

Femoral subtrochanteric fractures were classified according to the Seinsheimer classification, including 15 cases III A type, 7 cases III B type, and 30 cases IV type. After admission, patients were treated with manual reduction under continuous traction, and tibial tuberosity bone traction of the affected limb was performed. For patients with severe comorbidities, poor systemic conditions and severe osteoporosis, t-shaped shoes should be externally fixed. The surgical method was limited open reduction using prolonged PFNA combined with internal fixation with a ligation band. The duration from injury to operation ranged from 1 to 20 days, with an average of 3.87 ± 1.25 days.

### Surgical technique

The patient was placed in the supine position and anesthetized by general anesthesia. The lateral approach was used for all surgical approaches. A special orthopedic traction bed was used, and the lower limbs were fixed on the traction bed bracket after being protected by cotton pads. The undamaged limbs were fixed with the abduction and rotation position of the hip joint. The affected limbs were pulled by the traction bed, and the fractured end was reduced with proper adduction and rotation before being fixed. According to the degree of fracture fragmentation, the length and force line of the affected limb should first be restored.

With the fracture end as the center, a lateral longitudinal limited incision of 4–6 cm was made. After the epidermis and subcutaneous tissue were dissected, the deep fascia was separated to expose the posterior muscle space of the vastus lateralis muscle. Dissection of the origin of the vastus lateralis muscle should be made according to the need for surgical field exposure. To expose the fractured end, point reduction forceps should be used to reduce the bone mass directly under the naked eye. Excessive dissection of the medial bone should not be performed, and rough reduction of the medial bone should not be performed with holding forceps to avoid damaging the blood supply and decreasing the incidence of bone nonunion. After anatomical reduction of the fracture, the 1–2 femoral cerclage wire (titanium cable or steel wire) was fixed. In the case of a large femoral trochanter fracture, a cerclage wire was placed around the fracture block above the small trochanter, and the compression reduction was tightened to restore the integrity of the internal wall.

At the junction of the anterior 1/3 and posterior 2/3 of the greater trochanter, the needle was inserted into the distal medullary cavity through the fracture end after the opening of the hollow opener. C-arm X-ray fluoroscopy was employed to confirm that the needle was located in the medullary cavity.

After the medullary cavity reaming was guided by the guide needle, PFNA with appropriate length and diameter was selected and inserted into the medullary cavity. Under the guidance of the aiming frame, the spiral blade was driven into the proximal end. For young and middle-aged patients with good bone quality, the spiral blade can be inserted after reaming. For elderly and osteoporosis patients, the guide needle can directly insert the blade approximately 1.5 cm away from the subarticular cartilage bone to enhance the fastness of fixation. Two locking screws of appropriate length were placed in the distal femur.

### Postoperative functional follow-up

Postoperative follow-up recorded and assessed whether there was incision infection, deep vein thrombosis, varus coxa, and delayed union or nonunion of fractures. According to the Sanders functional rating system, the score was recorded as excellent (55–60 points), good (45–54 points), or poor (35–44 points).

### Establishment of a three-dimensional model of subtrochanteric fracture of the femur

**Fig. 3:**
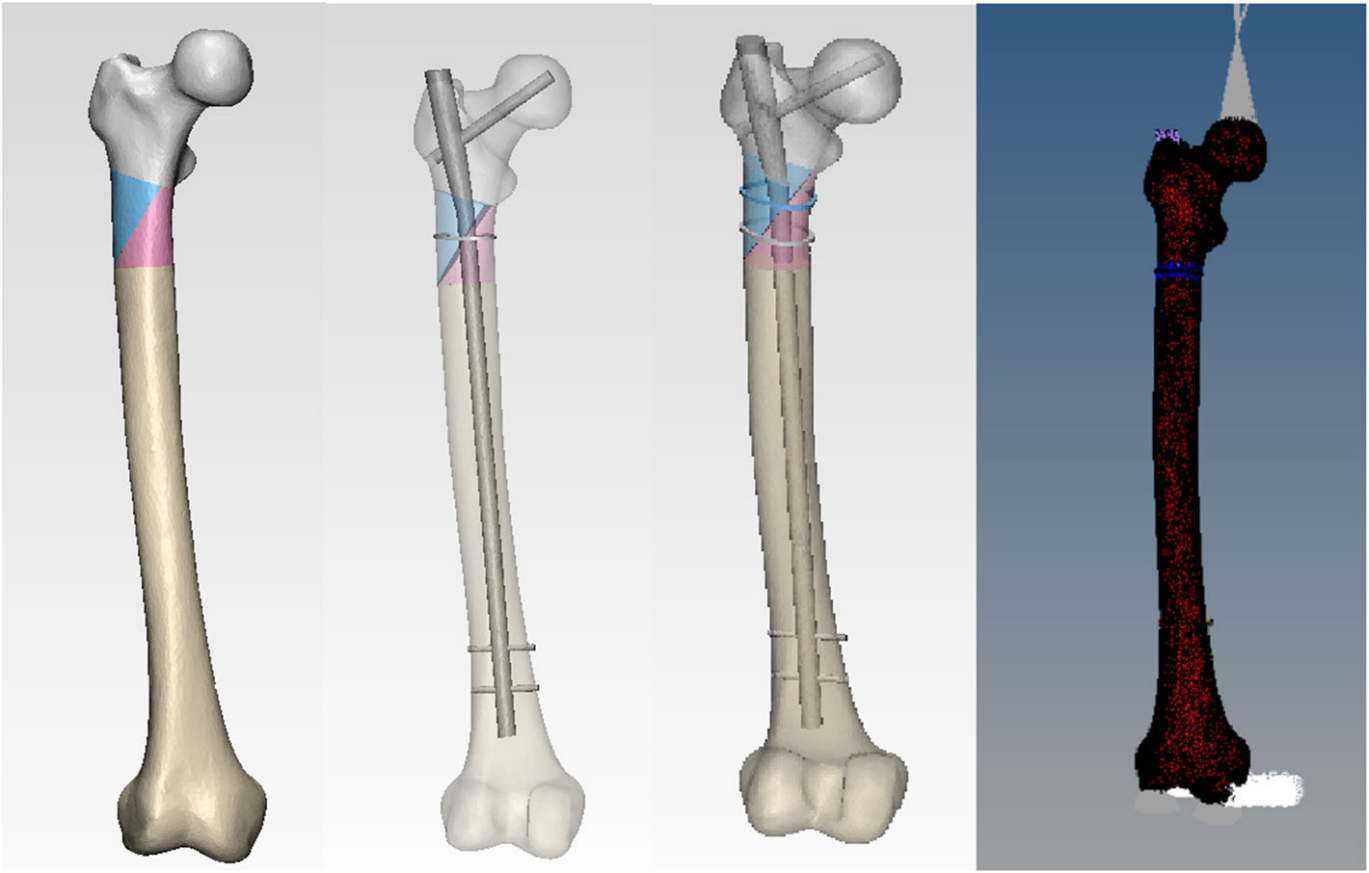
Seinsheimer IV fracture model is shown at the left. In the middle, the assembly drawing of Seinsheimer IV fracture model with PFNA and binding band fixation is presented. The right side is the loading of the fracture model. A 500 N load was applied to the femoral head, and the femoral medial and lateral condyles were fixed

The CT data extracted from patients were imported into Mimics 15.0 medical modeling software (Materialise, Belgium). A regional growing function was adopted to select two-dimensional femoral image data. The calculated 3D function was used to reconstruct the three-dimensional femoral model, which was output in STL format. The 3D femur model data were saved in STL format and imported into Geomagic Studio (Geomagic, USA). The model was sanded smooth with sandpaper and other features to remove the non-standard parts. With the interception function, the whole femur was cut into four fracture blocks according to the Seinsheimer IV fracture line and reassembled to ensure the complete alignment of the fracture lines. Using the assembly function, the PFNA model, the ring-type tie model, and the subtrochanteric fracture model were assembled to simulate the clinical internal fixation mode. The assembled models were saved separately and imported into 3-MATIC graphics processing software (Belgium Materialise) in STL format. The remainder of the function was used to reclassify and optimize the mesh. Finally, the optimized model was imported into Hypermesh 14.0 software (Altair, USA), and further finite element pretreatment steps such as assignment and load loading were carried out, as shown in Fig. 3.

### Material assignment and boundary condition

In Hypermesh 14.0 software, material properties are assigned to each component. The elastic modulus of the bone cortex is 17 GPa, and Poisson’s ratio is 0.33. The elastic modulus of cancellous bone is 5 GPa, and Poisson’s ratio is 0.33. The elastic modulus of the PFNA and ring-type tie bands is 110 MPa, and Poisson’s ratio is 0.3. The numbers of nodes and mesh of PFNA were 2602 and 29,697, respectively. The numbers of femur nodes and mesh were 12,906 and 120,679, respectively. The numbers of nodes and mesh of the cerclage wire model were 10 and 2710, respectively. The distal condylar articular surface of the femur was restrained, and a force of 500 N was applied vertically along the long axis of the femoral shaft with the femoral head as the loading point. The interface between the fracture blocks, PFNA, and femur was set as a friction coefficient of 0.3. The area between the cerclage wire and the femoral contact surface was set as rough.

## Results

### Clinical follow-up results

The operation times of all patients ranged from 60 to 130 min, with an average of 82 min, and the length of hospital stay was 5–37 days, with an average of 13.68 days. All patients were fully followed up for 12 to 36 months, with an average of 18.09 ± 4.63 months. The fracture healing time was 14.35 ± 2.67 weeks. Among them, deep vein thrombosis occurred in 3 cases after the operation. After active anticoagulant treatment, re-examination of lower extremity ultrasound indicated that the thrombosis had disappeared. Postoperative nonunion of fracture occurred in 1 patient, which was considered to be a serious fracture. The patient did not follow the doctor’s advice for early weight-bearing walking, and the fracture healed after reoperation with autologous iliac bone graft in combination with plate fixation. According to the Sanders score of hip joint function, there were 28 cases of excellent results (55–60 points), 22 cases of good results (45–54 points), and 2 cases of poor results (35–44 points), with an excellent and good rate of 96.15%. None of the patients had incision inflammation infection, internal fixation fracture, unequal limb length, coxal varus, or other complications.

### Maximum displacement and stress of a subtrochanteric fracture model

**Fig. 4:**
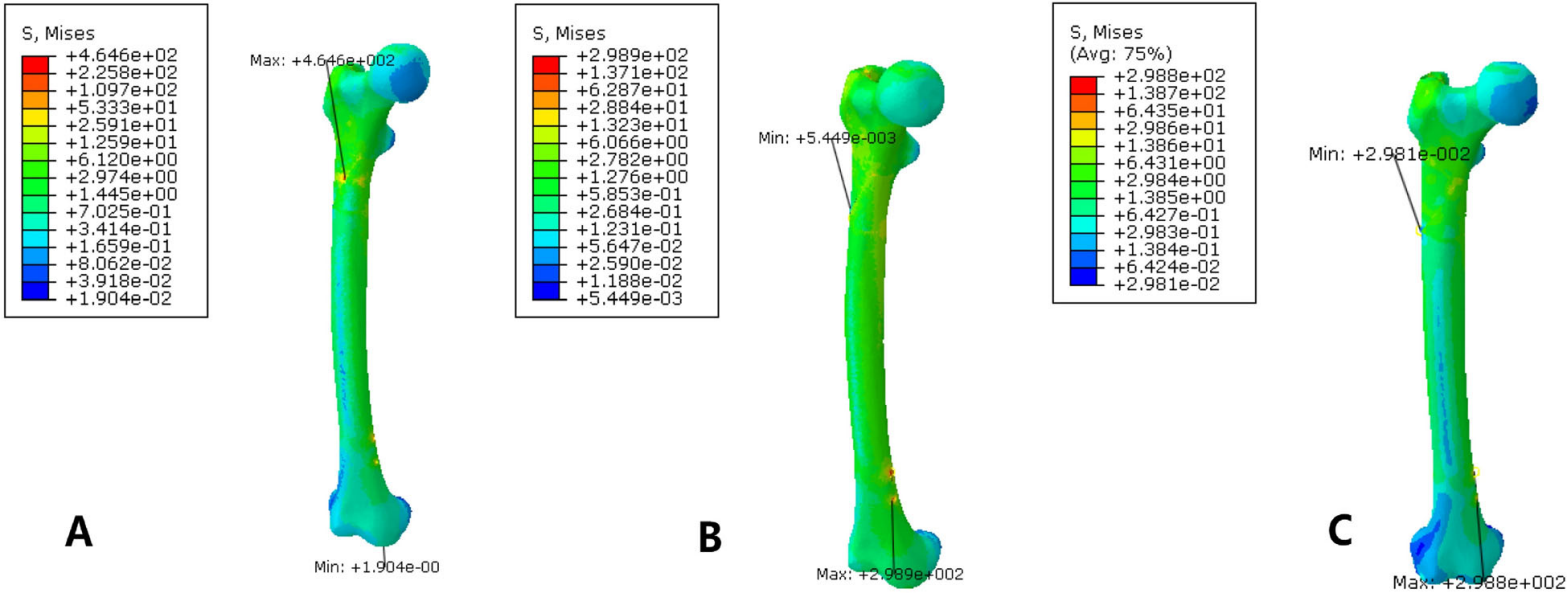
Stress distribution nephogram of femur. At the right is a displaced cloud image of a fracture fixation model. **a** is fixed with cerclage wire, while **b** is fixed with simple PFNA. **c** is for the fixation of double wires

**Fig. 5:**
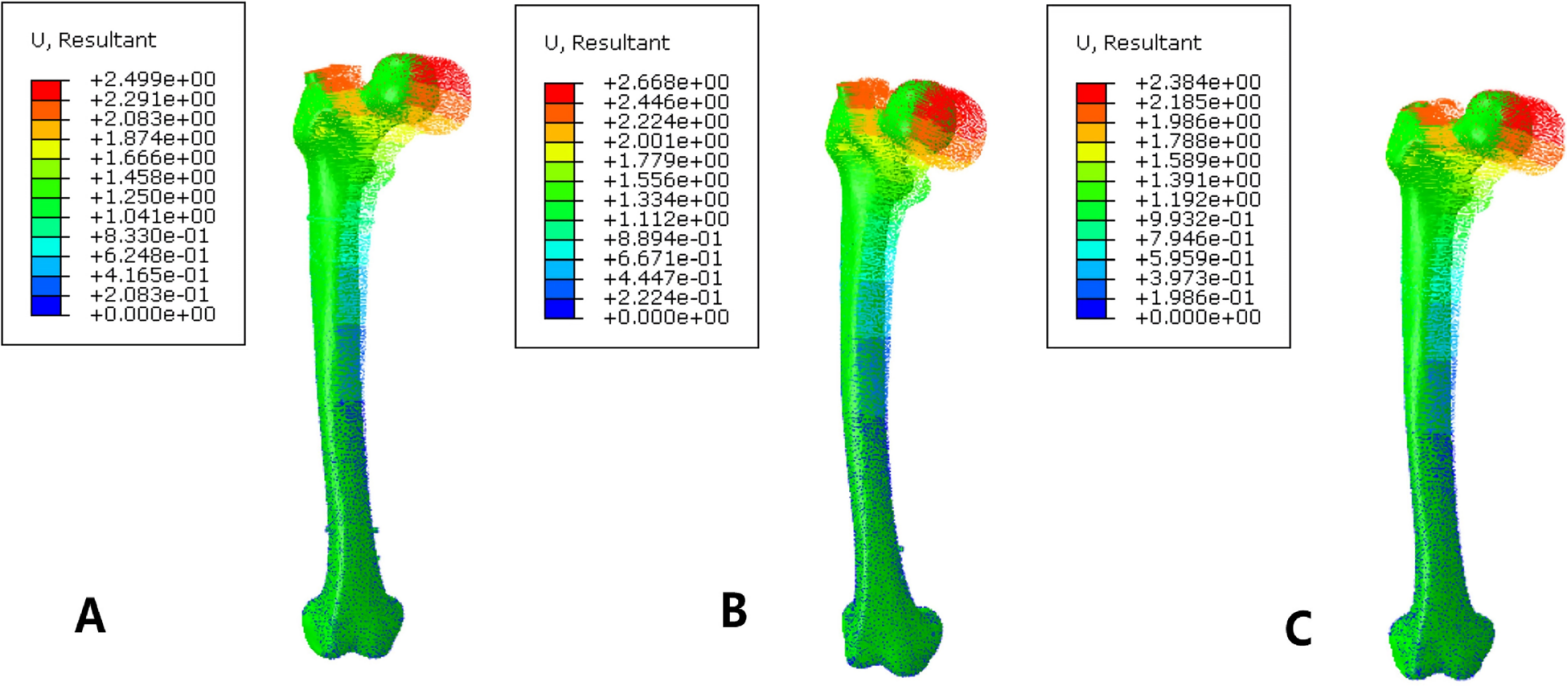
Nephogram of femoral displacement distribution. At the right is a displaced cloud image of a fracture fixation model. **a** is fixed with cerclage wire, while **b** is fixed with simple PFNA. **c** is for the fixation of double wires

The maximum stress on the femur of the PFNA model was 298.9 MPa, and the maximum stress was concentrated in the medial side of the screw hole at the most distal end. The maximum stress on the femur fixed by PFNA and cerclage wire was 464.6 MPa, and the maximum stress was concentrated in the position where the ligation and the femur contact each other. After the addition of the cerclage wire, the maximum stress of the femur increased significantly, and the maximum stress was transferred to the contact of the band and bone surface, as shown in Fig. 4. The maximum displacement of the femur of the PFNA fixation model was 2.668 mm, and the maximum displacement of the femur of the PFNA fixation model with cerclage wire was 2.499 mm. The maximum displacement of the double-wire fixation model was 2.38 mm. The displacement trend decreased from the proximal end to the distal end, as illustrated in Fig. 5. The displacement of the whole model decreased slightly after the addition of ligation and fixation, and the addition of another cerclage wire reduce the displacement compared to single wire fixation.

### Stress distribution and displacement of PFNA

**Fig. 6:**
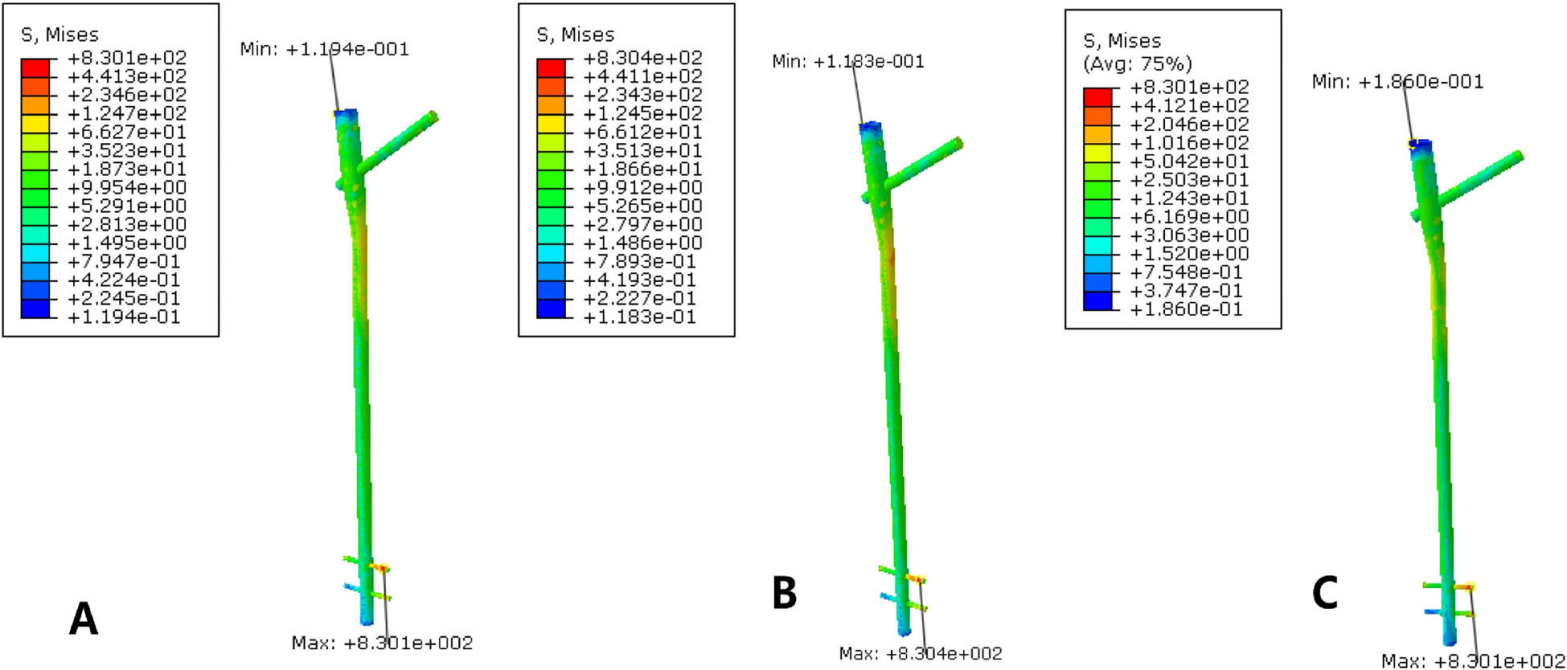
The stress distribution of PFNA in two internal fixation models. **a** is fixed with cerclage wire, while **b** is fixed with simple PFNA. **c** is for the fixation of double wires

The maximum stress on the PFNA model was 830.4 MPa, and the maximum stress was concentrated inside the distal penultimate screw. The maximum stress on the PFNA model was 830.1 MPa, and the maximum stress was also concentrated inside the penultimate screw at the distal end. The maximum stress of PFNA in the double-wire fixation model was 830.1 MPa, the same as that in the single-wire fixation model. The stress distribution of PFNA in the three groups of models was essentially the same, and the stress was concentrated at the angle of the main nail and the distal screw. The maximum stress of PFNA was slightly reduced after the addition of ligature and fixation, but the overall stress distribution did not change significantly, as shown in Fig. 6.

### The stress of the cerclage wire and its effect on the displacement of the fracture block

**Fig. 7:**
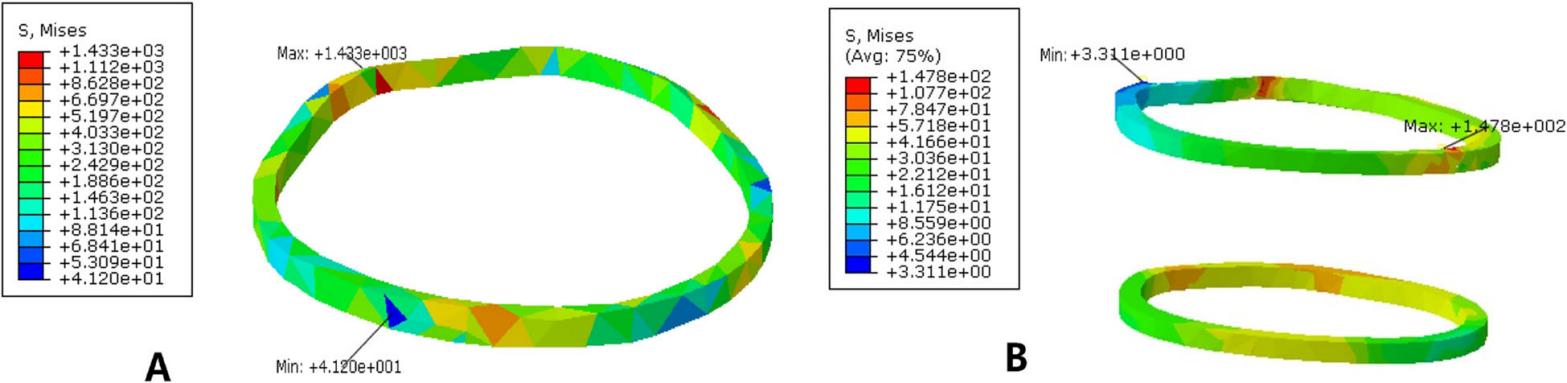
The stress distribution of the cerclage wire. The maximum stress of the single cerclage wire is 1433 MPa, which is located at the lateral position of the posterior femur of the wire. The minimum stress is 41.2 MPa and located at the lateral position of the anterior femur. The maximum stress of the double wire was fixed at 147.8 MPa at the anterior medial position of the femur, and the minimum stress was 3.31 MPa at the posterior lateral position of the femur

The maximum stress of the annular tie band was 1433 MPa, which was located at the back side of the tie band. The minimum stress was 41.2 MPa and was located at the lateral position of the anterior femur. The maximum stress of the double bands was 147.8 MPa at the anterior medial position of the femur, and the minimum stress was 3.31 MPa at the posterior lateral position of the femur, as shown in Fig. 7. The relative displacement of the fracture block in the PFNA plus cerclage wire fixation model was 0.04 ± 0.01 mm, the relative displacement of the PFNA double wire fixation model was 0.039 ± 0.05 mm, and the relative displacement of the fracture block in the PFNA alone fixation model was 0.082 ± 0.02 mm. The difference between the cerclage wire fixation group and the PFNA-alone fixation group was statistically significant (*P* < 0.001). There was no significant difference in the relative displacement between the single and double cerclage wire fixation.

## Discussion

Subtrochanteric fracture of the femur is a common lower limb fracture, especially in elderly patients and patients with high-energy trauma. In elderly patients, the cause of subtrochanteric fracture is mostly osteoporosis and low-energy injury. Subtrochanteric fractures of the femur were originally classified by Fielding [17], but the classification was based solely on the location of the fracture line lacks clinical significance.

The classification proposed by Russell-Taylor [18] mainly focuses on the piriformis fossa and the greater tubercle, which has important guiding significance for first-generation intramedullary nail treatment with the piriformis fossa as the entry point. However, with the evolution of internal fixation equipment, the opening of the new generation of intramedullary nails was gradually simplified to the opening at the top of the greater tubercle, and its practical significance gradually decreased. Seinsheimer classification was performed according to the number of fractured blocks and the location and shape of the fracture line. The fracture has been divided into five types. The medial cortex has great importance as the support structure and in its effect on the stability of the fracture, providing reference for the selection of clinical treatment. Therefore, the Seinsheimer classification of femoral subtrochanteric fractures has been widely used [19].

There are various fixation methods for subtrochanteric fractures of the femur, and appropriate internal fixators can be selected according to different fracture types. Currently, the main treatment method is the intramedullary nailing technique. Compared with the extramedullary fixation system, the intramedullary fixation system is less invasive, reducing intraoperative blood loss and reducing the risk of postoperative bone nonunion and postoperative infection.

Previous studies have indicated that the biomechanical results of intramedullary fixation were superior to those of other fixation methods [20]. For femoral subtrochanteric fractures, due to the destruction of the medial wall of the bone, shear stress and lateral tensile stress under the femoral trochanter area were produced, leading to fracture malunion, shortening of the limbs and hip varus risk. The unique advantages of intramedullary nails make them the preferred treatment for subtrochanteric fractures of the femur [7, 21]. The intramedullary nail has good mechanical stress-bearing performance with a short torque arm and small bending moment. In particular, when combined with an anti-spiral blade, the intramedullary nail has a strong anti-rotation ability. The load is shared both among the medial and lateral sides of the femur, which improves the overall stability of both the bone and the internal fixation. For Seinsheimer type III A and IV fractures, this type of fixation can significantly reduce the incidence of fracture malunion and internal fixation failure. At the same time, according to the situation of fracture healing, postoperative patients can be weight-bearing and active in the early stage. In this study, we selected extended PFNA intramedullary fixation, with limited open and anatomical reduction of the fracture end, using the cerclage wire to restore the integrity of the fracture, especially the complete supporting role of the medial bone [7, 22], to achieve good clinical efficacy.

Postoperative scores of Sanders hip joint function in 52 patients with subtrochanteric fracture of the femur were excellent in 28 patients and good in 22 patients, with an overall excellent and good rate of 96.15%. There was no inflammation of the incision, no fracture of the internal fixator, and no unequal limb complications.

The biomechanical strength of the femur internal fixation was enhanced after the addition of cerclage wire fixation. Many previous studies have reported satisfactory clinical outcomes of cerclage wire application in the treatment of subtrochanteric femur fracture. Hoskins et al. [23] conducted a 7-year retrospective review of all subtrochanteric fractures at a level 1 trauma center, and they concluded that in open reduction, a cerclage wire should be used as long as the fracture pattern allows. Karayiannis P [24] reviewed 465 patients with unstable intertrochanteric and subtrochanteric femoral fractures. It was found that cerclage cables/wires can augment fixation in subtrochanteric fractures with potential benefits including improving quality of reduction. However, evidence for their use in intertrochanteric fractures is much more contentious and it should be used where a definite improvement in reduction can be obtained. A prospective cohort study conducted by Codesido P [25] revealed that better reduction is achieved when using cerclage wires for fragility subtrochanteric fractures, and better outcomes in terms of life quality were observed in the cerclage group.

Finite element analysis showed that the maximum displacement of the femur of the PFNA fixed model was 2.668 mm, and the maximum displacement of the femur of the PFNA fixed model with cerclage wire was 2.499 mm, which was slightly reduced. Moreover, the relative slip between fracture blocks was reduced after the addition of cerclage wire ligation, suggesting that ligation can effectively increase the overall stability of the fracture model, and the fixation of ligation can also stabilize the relative slip between fracture blocks. Conversely, the maximum stress of PFNA decreased from 830.4 MPa to 830.1 MPa after the addition of cerclage wire. The addition of ligation reduces the stress load of PFNA intramedullary fixation to a certain extent, which is conducive to the maintenance of intramedullary fixation and reduces the risk of stress concentration.

Local stress concentrations in the cerclage wire may increase the risk of failure. The maximum stress of the cerclage wire is 1433 MPa, which is located at the back side of the belt. The maximum stress of PFNA was 830.4 MPa in the simple fixed PFNA model. The maximum stress of the cerclage wire was much higher than that of PFNA, which was doubled after the addition of PFNA to the ligature.

The greater stress applied to the femoral bone surface increase the failure risk of the ligation itself; thus, fracture of the cerclage wire may occur after the operation [7, 22]. The maximum stress of the cerclage wire was reduced to 147.8 MPa after another cerclage wire was added for fixation. Therefore, in clinical applications, the stress concentration can be dispersed by appropriately increasing the number of wires. However, considering the large tissue dissection that affects fracture healing, it is common to add up to two additional bands clinically.

In conclusion, this study confirmed that PFNA combined with cerclage wires in the treatment of unstable subtrochanteric fractures was easy to perform, with satisfactory fracture reduction, stable fixation, and good postoperative limb function recovery. At the same time, the biomechanical effect of fixation with cerclage wires on fracture fixation is explained through finite element analysis, which provides a reference for clinical treatment.

## Data Availability

The datasets used and analyzed during the current study are available from the corresponding author on reasonable request.

## Abbreviations

STL: Standard Triangle Language
PFNA: Proximal Femoral Nail Anti-rotation
